# Gut microbiota and epigenetic age acceleration: a bi-directional Mendelian randomization study

**DOI:** 10.1007/s40520-024-02877-6

**Published:** 2024-11-29

**Authors:** Han Xu, Ouyang Li, Dayoung Kim, Zhijun Bao, Fan Yang

**Affiliations:** 1 Department of Gerontology, Huadong Hospital Affiliated to Fudan University, Shanghai, China; 2 Shanghai Key Laboratory of Clinical Geriatric Medicine, Shanghai, China

**Keywords:** Gut microbiota, Epigenetic age acceleration, Ageing, Mendelian randomization, Causal relationship

## Abstract

**Background:**

The gut microbiota is closely related to aging, but the genetic relationship between gut microbiota and aging has not been well investigated. The aim of the study was to explore the association of microbiota with epigenetic age acceleration (EAA) using the Mendelian randomization.

**Method:**

The independent genetic instruments of gut microbiota were obtained from MiBioGen consortium and the Dutch Microbiome Project. EAA data were derived from genome-wide association study. To assess the causal relationship between gut microbiota and EAA, we applied four different methods of Mendelian Randomization (MR) analysis: the inverse variance weighted method (IVW), the MR-Egger regression, the weighted median analysis (WMA), and the weighted mode. Furthermore, sensitivity analyses were conducted to evaluate heterogeneity and horizontal pleiotropy.

**Results:**

We identified potential causal associations between 12 bacterial taxa and EAA (*P*_*IVW*_ and *P*_*WMA*_ < 0.05). Among them, species Holdemania_unclassified (OR: 1.31, 95% CI: 1.13–1.52, *P =* 0.0004) retained a strong positive association with GrimAge acceleration. Family Acidaminococcaceae (OR: 0.64, 95% CI: 0.44–0.93, *P =* 0.019) and family Clostridiaceae1 (OR: 0.69, 95% CI: 0.49–0.97 *P =* 0.031) were negative association with GrimAge acceleration. Reverse MR analyses indicated that EAA was associated with 6 bacterial taxa in IVW and WMA. Among them, a strong inverse association was found between Phenoage acceleration and genus Turicibacter (OR: 0.928, 95%CI: 0.888–0.971, *P*_*IVW*_ and *P*_*WMA*_ < 0.001).

**Conclusion:**

Our study implicates the potential causal effects of specific microbiota on EAA, potentially providing novel insights into the prevention aging through specific gut microbiota.

**Supplementary Information:**

The online version contains supplementary material available at 10.1007/s40520-024-02877-6.

## Introduction

Ageing is a complex, multifaceted process leading to widespread functional decline that affects every organ and tissue. The accumulation of aging cells in organisms, accompanied by the decline of biological functions and a series of prominent features, is including genetic and epigenetic changes [1]. The aging process can present as reduced physical activity, weakened digestive function, decreased nutrient absorption, and lowered immune response [2, 3]. Therefore, it is essential to identify the causes of accelerated aging and to prevent aging. In recent years, with rapid development of genetics and molecular biology techniques, epigenetic age has become the gold standard measure of biological aging [1, 4, 5]. DNA methylation age, also known as epigenetic clock, constructed based on DNA methylation data, can accurately assess the biological age of the human body and has been applied in the study of aging-related diseases [6, 7]. Epigenetic age acceleration (EAA) is the regression residual between DNA methylation age and chronological age, independent of the actual age, reflecting the senescence process [8, 9]. Numerous measures of EAA have been developed, each measuring unique aspects of the aging process, and include, among others: intrinsic epigenetic age acceleration (IEAA), HannumAge acceleration (HannumAA), GrimAge acceleration (GrimAA) and PhenoAge acceleration (PhenoAA). Effective identification and control of factors that accelerate aging will help prevent premature death, extend healthy life expectancy and improve life quality.

The aging process is regulated by multiple factors, and recent studies have found a close relationship between the gut microbiota and aging [10, 11]. The gut microbiota is a dynamic and complex community of ecological microbes, inhabiting the human intestine, contributing to multiple metabolism process. With the decline of digestive functions, the gut microenvironment undergoes varying degrees of changes, indirectly influencing the nutritional intake of older adults and causing alterations in the gut microbiota [12, 13]. Furthermore, previous research has shown that certain characteristic microbial composition undergoes changes during unhealthy aging processes [14, 15]. The microbiota accelerates age-related methylation drift in the colon when compared with germ-free mice [16]. Fecal transplantation from aged mice into young mice has been found to increase intestinal inflammation [17]. However, there are also studies indicating that fecal microbiota transplantation from healthy aging mice can improve gut barrier function and enhance cognitive function [18]. Therefore, it is necessary to identify the causality between specific gut microbiota and aging.

Limited by the quality of evidence, possible potential reverse causality and residual confounding, observational studies have been almost unable to identify a causal association between microbiota and aging. Mendelian randomization (MR) study is a genetics-based epidemiological approach using large population data that allows causal inference at the genetic level by replacing the phenotype with a specific genetic variant [19]. Because of these advantages, MR studies can overcome the limitations of observational studies and produce more reliable conclusions. Previously, one MR study demonstrated a causal link between genetically predicted epigenetic age and parental lifespan in individuals of European descent [20]. Besides, research has also found gut microbiota components that are causally associated with sarcopenia [21]. However, there is still no such kind of studies that performed a comprehensive analysis focusing on the relationship between EAA and gut microbiota. Therefore, our study is demonstrating MR studies relating biological aging to gut microbiota.

## Method

### Study design

**Fig. 1:**
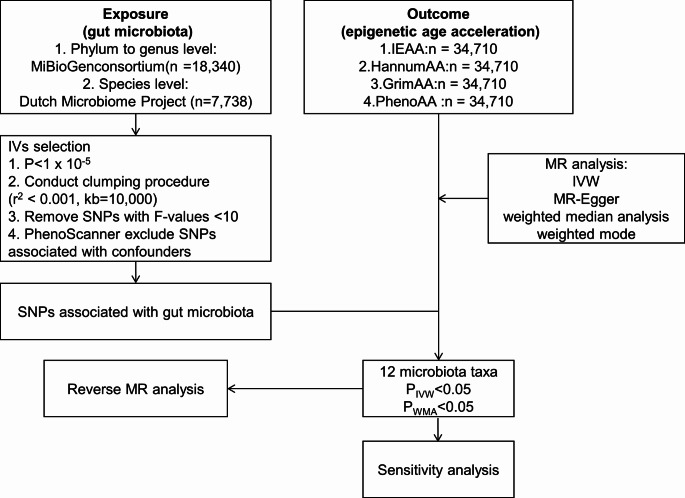
The study design and workflow of the present MR study. IEAA, intrinsic epigenetic age acceleration; HannumAA, HannumAge acceleration; PhenoAA, PhenoAge acceleration; GrimAA, GrimAge acceleration; IVs, instrumental variables; MR, Mendelian randomization; SNP, single nucleotide polymorphism; IVW, Inverse-variance-weighted;

The study design of the present two-sample MR analysis is shown in Fig. 1. To reliably infer the potential causality of gut microbiota with EAA using MR approach, we tried to meet three key assumptions of MR analysis. First, the instrumental variables (IVs) are correlated with gut microbiota. Second, IVs are unrelated to confounders influencing this association. Third, IVs influence the EAA only through gut microbiota [22].

### Data sources and instruments selection

The characteristics of data in this study were presented in Supplemental table S1. The genetic data of human gut microbiome at the phylum to genus level were acquired from the MiBioGen study (data from https://mibiogen.gcc.rug.nl/) comprising 24 population-based cohorts with 18,340 participants [19]. The majority of participants in the study were of European descent (*N* = 13,266). Subsequently, taxonomic classification was performed utilizing direct taxonomic binning. Profiling of microbial composition was achieved through targeted sequencing of the, V3-V4, and V1-V2 regions of the 16 S rRNA gene. A total of 211 bacterial groups were included in this study. Our study excluded 15 unknown bacterial taxa, leaving 196 bacterial taxas, encompassing 119 genus, 31 families, 20 orders, 16 classes, and 9 phyla. The genetic data of microbiota species were derived from the Dutch Microbiome Project which investigated the composition and function of the gut microbiome in 7,738 participants of European ancestry [23]. Of these, total 101 species were identified. To obtain more comprehensive results, IVs that attained locus-wide significance (*P* < 1 × 10^− 5^) were selected. In parallel, single-nucleotide polymorphisms (SNPs) in linkage disequilibrium were excluded by the PLINK clumping method (r^2^ < 0.001, kb = 10,000). Then, SNPs with F-statistics [formula: R^2^/K × (N-K-1)/ (1-R^2^)] < 10 were removed [24]. According to previous studies, muscle mass detection by bioelectrical impedance is related to aging [25], in addition to smoking [26], drinking, obesity [27], and some diseases such as hypertension [28], Crohn’s disease [29], Parkinson’s [27], asthma [30], and psoriasis [31], which are also associated with epigenetic aging. Therefore, we searched the PhenoScanner website (http://www.phenoscanner.medschl.cam.ac.uk/) for phenotypes associated with gut microbiota-related SNPs and removed 14 SNPs associated with confounders (Supplemental table S3). A total of 296 bacterial taxa and 3,537 SNP were included in the MR analysis.

To measure biological aging, genome-wide association study (GWAS) of European-ancestry participants (*n* = 34,710) of four types of EAAs reported by McCartney et al. were exploited, which integrated data from 29 studies [32]. Four separate epigenetic clocks: IEAA, HannumAA, GrimAA, andPhenoAA were evaluated. IEAA, derived from the Horvath clock, was developed to alleviate the influence of varied blood components. The HannumAA is similar to the remaining clocks, was strictly derived from blood leukocytes. To better link epigenetic alterations with age-related outcomes, PhenoAA and GrimAA were developed, which were usually called the second generation of epigenetic clocks. The PhenoAA was calibrated using a weighted average of chronological age and 9 clinical laboratory values [33], whereas GrimAA was developed by regressing time to death on a composite of 7 DNA methylation scores calibrated for plasma clinical biomarkers associated with mortality [34].

Due to the fact that the *P*-value threshold of 5e-08 resulted in a limited number of valid instruments to choose from, we selected a *P*-value threshold of 1e-05 to screen more valid instruments. The detailed information of the instruments used for the MR analysis is provided in Supplemental Table S2, and the instruments selected by the *P*-value threshold of 5e-08 are listed in Supplemental Table S7.

### MR analysis

Four methods were used to assess the relationship between gut microbiota and EAA. The inverse variance weighted (IVW) approach was used as the principal MR method, which incorporates the Wald estimator and Delta method of SNPs to estimate the effect, with the intercept constrained to zero [35]. We chose the IVW fixed-effect model used in the absence of any potential horizontal multiplicity heterogeneity and random-effect model used in the presence of heterogeneity. Three other methods, namely the MR-Egger regression, the weighted median analysis (WMA), and the weighted mode were used as secondary references. The MR-Egger approach assumes that all SNPs were invalid instrumental variables and defaulted to the presence of an intercept term [36]. The weighted median analysis approach uses the median of the IVW ratio estimates, and it is effectively applicable when the pleiotropy was less than 50%, which improved the accuracy of the results to some extent [37]. The weighted mode approach uses the mode of the IVW ratio estimates, but it is less powerful than the IVW approach [38]. It gives consistent estimates if the maximum weight comes from valid genetic variants. The above methods base on the examining violations of exclusion restriction, especially horizontal pleiotropy. The methods rely on different assumptions that can only be partially tested. Therefore, it is beneficial to compare the results of different methods, as this can reveal a variety of potential threats to the IV-assumptions.

To obtain a more rigorous explanation of causality, the results of IVW test corrected by FDR (false discovery rate control). Considering that the FDR corrected by the number of microbes would be too stringent, we also added Bonferroni method to establish multiple testing significance thresholds at different taxonomic levels according to previous research [40–42]. The significance thresholds were calculated based on the number of bacteria under each taxonomic level. The thresholds for each level were as follows: 5.6 × 10^− 3^ (0.05/9) for phylum, 3.1 × 10^− 3^ (0.05/16) for class, 2.5 × 10^− 3^ (0.05/20) for order, 1.6 × 10^− 3^ (0.05/31) for family, 4.2 × 10^− 4^ (0.05/119) for genus, and 5.0 × 10^− 4^ (0.05/101) for species. *P* values reaching nominal significance (*p* < 0.05) were considered to have nominal potential causal effects. Finally, a reverse MR analysis was performed to assess the possibility of reverse causality between genetically predicted EAA and gut microbiota.

### Sensitivity analysis

To inspect the heterogeneity and validity of our results, the following sensitivity analyses were performed sequentially: (1) The MR-Egger regression tests were performed to determine the directional pleiotropy if the intercept term had statistical significance. (2) Cochran’s Q tests were calculated to quantify the heterogeneity among the selected SNPs. (3) Mendelian randomization of pleiotropy residuals and outliers (MR PRESSO) was calculated to detect outliers for pleiotropy bias. (4) A leave-one-out sensitivity analysis was used to see if there was a statistical difference before and after excluding each SNP one by one. (5) MR Steiger tests were operated to distinguish the directivity of the impact of exposure on the outcome, with a “TRUE” result signaling that the association is in expected direction. Through the above detections, we can further confirm the accuracy and robustness of the MR results.

The threshold for significance was *P* = 0.05. All statistical analysis was conducted using R, Version 4.3.1 with the two-sample MR and MR PRESSO packages.

## Results

### Overview

**Fig. 2:**
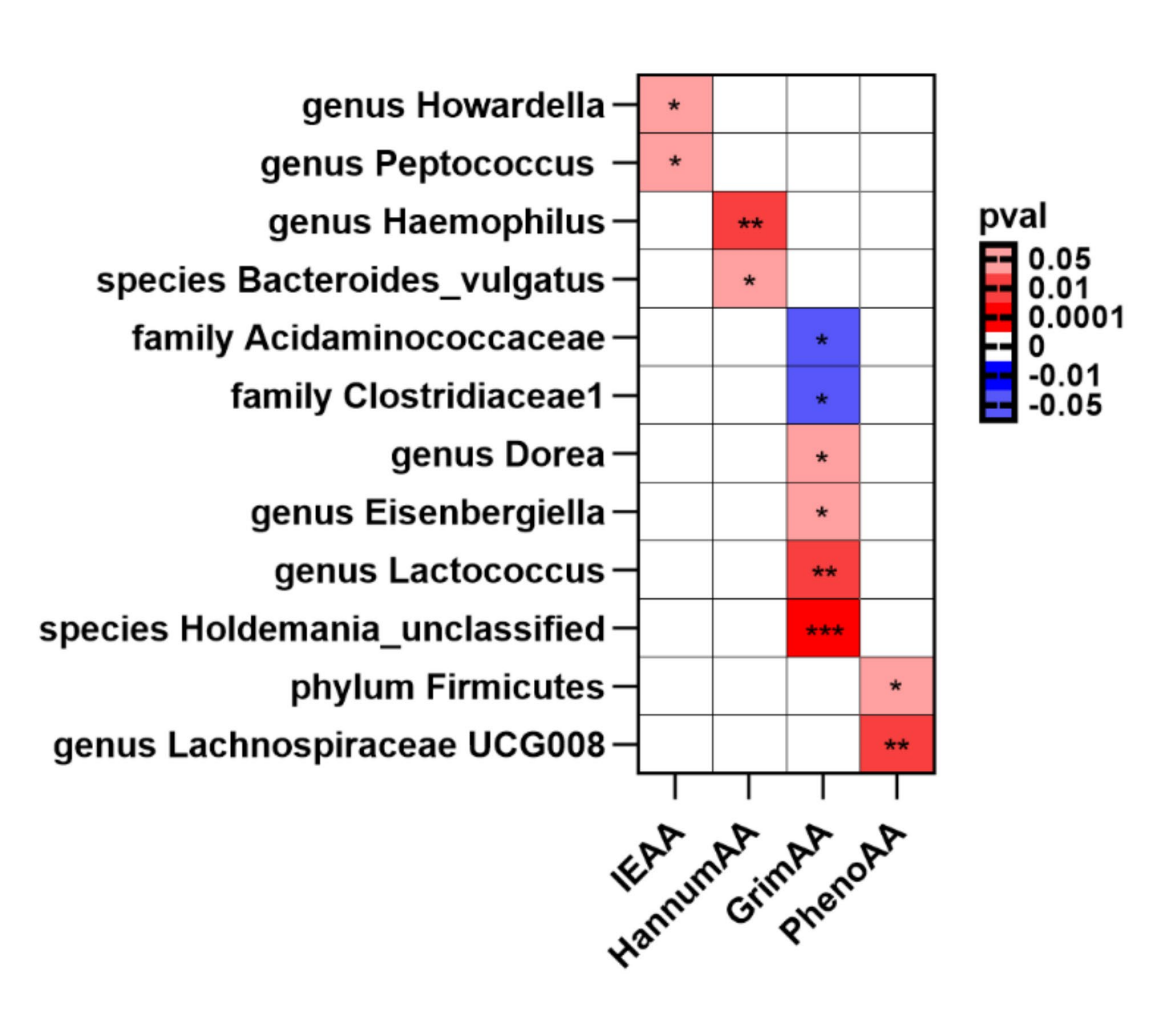
The potential causal relationship between gut microbiota and EAA in the IVW method (*P*_*IVW*_ and *P*_*WMA*_ < 0.05). The negative values represent negative correlations. Red represents the microbiota positive associated with EAA, blue represents the microbiota negative associated with for EAA, and white represents no causal microbiota for EAA. ***represent *P*_*IVW*_<0.001, ** represent *P*_*IVW*_<0.01, * represent *P*_*IVW*_<0.05

**Fig. 3:**
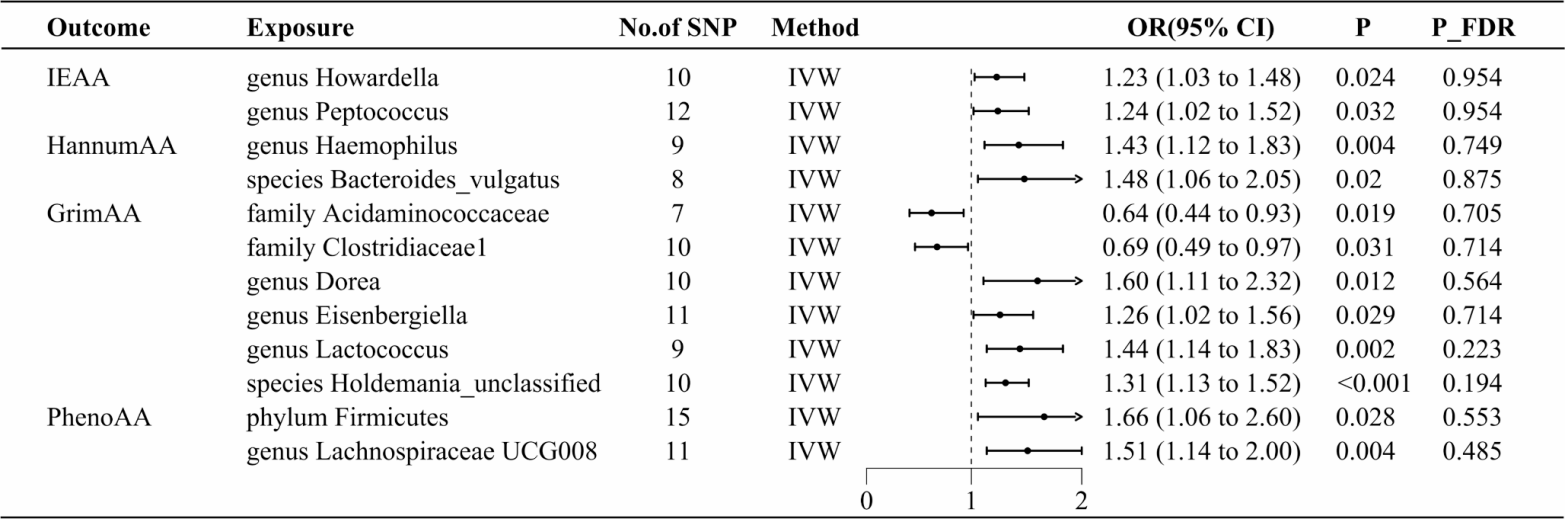
Forest plot to visualize the causal effect of significant gut microbiota on the risk of EAA of MR analysis

A total of 3,059 eligible SNPs from 296 microbial taxa were finally included in this analysis. Details of IVs are listed in Supplemental Table S2. The IVW method and WMA identified 12 microbial taxa associated with EAA (Fig. 2). The scatter plots of the associations of these 12 microbial taxa with the corresponding EAA are shown in Supplemental figure S1–S4. The associations between genetically predicted 12 microbial taxa and EAA are shown in Fig. 3.

### Gut microbiota and IEAA

This study identified 4 different microbial taxa with IEAA, genus Howardella (OR: 1.23, 95% CI: 1.03–1.48, *P =* 0.024) and genus Peptococcus (OR: 1.24, 95% CI: 1.02–1.52, *P =* 0.032) were associated with a higher risk of IEAA (Fig. 3). In addition, the leave-one-out method (Supplemental Figure S5) showed that results were stable to the outcomes of this MR analysis.

### Gut microbiota and HannumAA

A higher genetically predicted genus Haemophilus (OR: 1.43, 95% CI: 1.12–1.83, *P =* 0.004) and species Bacteroides_vulgatus (OR: 1.48, 95% CI: 1.06–2.05, *P =* 0.02) were associated with an increase in HannumAA (Fig. 3). In addition, the leave-one-out method (Supplemental Figure S6) showed that results were stable to the outcomes of this MR analysis.

### Gut microbiota and GrimAA

Between GrimAA, a causal correlation was found in six microbiotas. A higher genetically predicted family Acidaminococcaceae (OR: 0.64, 95% CI: 0.44–0.93, *P =* 0.019) and family Clostridiaceae1 (OR: 0.69, 95% CI: 0.49–0.97, *P* = 0.031) were associated with a decrease risk in GrimAA (Fig. 3). Whereas, genus Dorea (OR: 1.60, 95% CI: 1.11–2.32, *P =* 0.012), genus Eisenbergiella (OR: 1.26, 95% CI: 1.02–1.56, *P =* 0.029), genus Lactococcus (OR: 1.44, 95% CI: 1.14–1.83, *P =* 0.002) and species Holdemania_unclassified (OR: 1.31, 95% CI: 1.13–1.52, *P =* 0.0004) were associated with an increase in GrimAA (Fig. 3). Finally, the leave-one-out method (Supplemental figure S7) showed that results were stable to the outcomes of this MR analysis.

### Gut microbiota and PhenoAA

Genetically predicted 2 microbiotas (Fig. 3) were associated with an increased risk of PhenoAA, including phylum Firmicutes (OR: 1.66, 95% CI: 1.06–2.6, *P =* 0.028) and genus Lachnospiraceae UCG008 (OR: 1.51, 95% CI: 1.14-2, *P =* 0.004) In addition, the leave-one-out method (Supplemental Figure S8) showed that results were stable to the outcomes of this MR analysis.

### Bonferroni correction and sensitivity analysis

The Bonferroni correction indicated that only species Holdemania_unclassified (OR: 1.31, 95% CI: 1.13–1.52, *P =* 0.0004) retained a strong positive association with GrimAA. Furthermore, no obvious heterogeneity was found according to results from Cochrane’s Q test and there was no evidence of directional pleiotropic effects from MR-egger regression (Supplemental table S4). The MR PRESSO test did not detect any outliers (Global Test *P*-value>0.05). Likewise, steiger test results showed the right causal direction, which is that IVs influence exposure factors first and then influence outcomes through exposure factors (Supplemental table S4).

### Reverse MR analysis

The selection criteria for IVs in reverse MR must first ensure their association with EAA. Second, IVs are unrelated to confounders influencing this association. Third, IVs influence the gut microbiota only through EAA. Specifically, we used SNPs with *P*-values < 5e-8 as instrumental variables (IVs) and excluded SNPs associated with confounders. We further ensured that the selected SNPs had high F-statistics values, confirming their relevance to the exposure. After a series of IV screening steps, 20 IEAA associated-SNPs, 3 HannumAA-associated SNPs, 8 PhenoAA-associated SNPs were identified IVs. For GrimAA, reverse MR analysis was not performed because we found < 3 SNPs in GrimAA associated with gut microbiota. The Details of IVs for reverse MR are listed in Supplemental Table S5.

We found that 3 microbial taxa were associated with IEAA, and 3microbial taxa were associated with PhenoAA by both the *P*_*IVW*_ and *P*_*WMA*_ <0.05 (Supplemental Table S6). Specifically, IEAA was positively correlated with genus Marvinbryantia (OR 1.037, 95% CI 1.006–1.070, *P =* 0.020), and PhenoAA was positively correlated with genus Oscillibacter (OR 1.069, 95% CI 1.022–1.118, *P =* 0.003). Besides, IEAA was negatively correlated with genus Ruminococcaceae UCG011 (OR 0.949, 95% CI 0.903–0.998, *P =* 0.040) and species Bacteroides_eggerthii (OR 0.940, 95% CI 0.892–0.991, *P =* 0.021). Likewise, PhenoAA was negatively correlated with genus Turicibacter (OR 0.928, 95% CI 0.888–0.971, *P =* 0.001) and species Holdemania_unclassified (OR 0.892, 95% CI 0.828–0.962, *P =* 0.003).

## Discission

Through MR analysis of gut microbiota and epigenetic age acceleration (IEAA, HannumAA, GrimAA, PhenoAA), we identified potential causal associations between 12 microbiota and EAA (*P* < 0.05 in IVW and WMA). In our research, we observed potential associations between certain microbial families and genera with the risk of EAA. Specifically, family Acidaminococcaceae and family Clostridiaceae1 appeared to be associated with a decreased risk of EAA. Conversely, genus Howardella, genus Peptococcus, genus Haemophilus, species Bacteroides_vulgatus, genus Dorea, genus Eisenbergiella, genus Lactococcus, species Holdemania_unclassified, phylum Firmicutes, and genus Lachnospiraceae UCG008 showed potential associations with an increased risk of EAA.

Holdemania is a Gram-positive, strictly anaerobic and non-spore-forming genus from the family of Erysipelotrichaceae. Even there is no direct evidence linking the species Holdemania to aging, previous studies have linked Holdemania with a number of diseases. Holdemania is enriched in the feces of Parkinson’s disease patients [42], while its abundance is higher in those with higher alcohol consumption [43]. Besides, Holdemania is linked to impaired lipid metabolism and negatively correlated with skeletal muscle mass in elderly women [44]. Furthermore, in a Mendelian analysis analysis the relationship between gut microbiota and atrial fibrillation, Holdemania was found to be associated with a higher incidence of atrial fibrillation, and this association was mediated by body mass index regulation [45]. Previous research has also indicated that Holdemania is involved in mucin degradation [46], which can lead to intestinal barrier damage and trigger systemic inflammation and may associated with ageing. In our MR analysis, we found that species Holdemania has a strong positive association with GrimAA.

Moreover, our results also indicate the gut microbiota decreased epigenetic age acceleration, family Acidaminococcaceae and family Clostridiaceae1 were strongly associated with the lower risk. Acidaminococcaceae is a family of bacteria that belongs to the phylum Firmicutes. Members of this family are Gram-positive found in the gastrointestinal tract of humans and animals [47]. Previous studies have shown that Acidaminococcaceae is significantly reduced in the feces of patients with hypertension and coronary heart disease [48]. Similarly, in patients with irritable bowel syndrome (IBS) and children with autism spectrum disorder (ASD), the abundance of Acidaminococcaceae in the feces is also significantly decreased [49, 50]. Acidaminococcaceae is also associated with the production of short-chain fatty acids. In patients with renal anemia, the concentration of Acidaminococcaceae in the feces increases and the expression of inflammatory factors decreases after treatment [51]. Clostridiaceae1is considered to be a bacterium that produces short-chain fatty acids. It has been observed to be reduced in patients with stroke [52]. After treatment with antioxidant drugs, the abundance of Clostridiaceae1 significantly increases in the gut of aging individuals [53]. However, there are also studies suggesting that Clostridiaceae1 may be a risk factor for type 2 diabetes [54]. Although research on these bacterial taxa is limited, their impact on overall health is still worthy of investigation.

The major advantage of our study is that we comprehensively analyzed the potential causalities of 296 microbial taxa and four different instruments for epigenetic age acceleration. The gut microbiota data from the MiBioGen consortium and Dutch Microbiome Project, which are currently the largest database with all microbial level from phylum to species. Our study has several strengths. Firstly, Mendelian randomization employs genetic variation as instrumental variables, which inherently minimizes confounding factors to a great extent. Secondly, as the latest GWAS on gut microbiota and epigenetic age acceleration to date, our study has sufficient statistical power to detect potential causal effects. Our findings suggest that further investigation into the screening of gut microbiota in individuals genetically predisposed to EAA may be warranted. Nonetheless, our study had some limitations. Firstly, the sample size of the GWAS summary data for species-level gut microbiome taxa was the largest one to date, it may not have been sufficient to detect all potential causal relationships given the high heterogeneity of the gut microbiota between populations. Secondly, since SNPs with *p* < 5 × 10^− 8^ were too limited, we selected SNPs with *p* < 1 × 10^− 5^ as IVs. To obtain reliable IVs, we performed a series of IV screening steps, including excluding SNPs with F < 10 to avoid weak IVs bias and searching all SNPs in PhenoScanner to avoid confounding effects. Third, when extrapolating our findings to other racial populations should be cautiously, as our research participants were of European ancestry. Fourth, this MR is a correlation analysis of gut microbiota and EAA without explaining the mechanism. Therefore, further research with larger GWAS datasets is warranted to validate our findings. Fifth, our research should ideally be applied using non-overlapping datasets for obtaining genetic associations with the exposure and the outcome to reduce weak instrument bias [55]. However, overlap between participants was unavoidable for some traits because the MiBioGen consortium was the largest available published GWAS of microbiota. Such as NTR (the Netherlands Twin Registry), which contains 6.8% participants in EAA group, the Rotterdam Study 3 contains 2.3% participants in EAA group, and TwinsUK contains 1.1% participants in EAA group [19]. Sixth, while we strive for uniformity throughout population sources, a small part of the gut microbiota data was obtained from multiple race sets, which may have biased our estimates. Finally, in terms of statistical methods, since the number of microbial taxas after FDR correction was too small, we chose the Bonferroni correction based on previous literature.

In conclusion, our findings suggest potential associations between gut microbiota and EAA. Our research identifies specific microbiota using genetic prediction, which may offer promising avenues for further exploration as potential biomarkers for early aging prevention.

## Electronic supplementary material

Below is the link to the electronic supplementary material.

Supplementary Material 1

Supplementary Material 2

## Data Availability

No datasets were generated or analysed during the current study.
