# Supplementary material for: Gut microbiota and epigenetic age acceleration: a bi-directional Mendelian randomization study
Source: Aging Clin Exp Res. 2024 Nov 29;36(1):227. doi: 10.1007/s40520-024-02877-6 (PMC11607049; doi:10.1007/s40520-024-02877-6)

**Supplemental data**

**Supplemental figure S1** Scatter plot for gut microbiota affecting IEAA risk

**Supplemental figure S2** Scatter plot for gut microbiota affecting HannumAA risk

**Supplemental figure S3** Scatter plot for gut microbiota affecting GrimAA risk

**Supplemental figure S4** Scatter plot for gut microbiota affecting PhenoAA risk

**Supplemental figure S5** Leave-one-out test for gut microbiota affecting IEAA

**Supplemental figure S6** Leave-one-out test for gut microbiota affecting HannumAA

**Supplemental figure S7** Leave-one-out test for gut microbiota affecting GrimAA

**Supplemental figure S8** Leave-one-out test for gut microbiota affecting PhenoAA

**Abbreviations:** IEAA, intrinsic epigenetic age acceleration; HannumAA, HannumAge acceleration; GrimAA, GrimAge acceleration; PhenoAA, PhenoAge acceleration

**Supplemental figure S1** Scatter plot for gut microbiota affecting IEAA risk


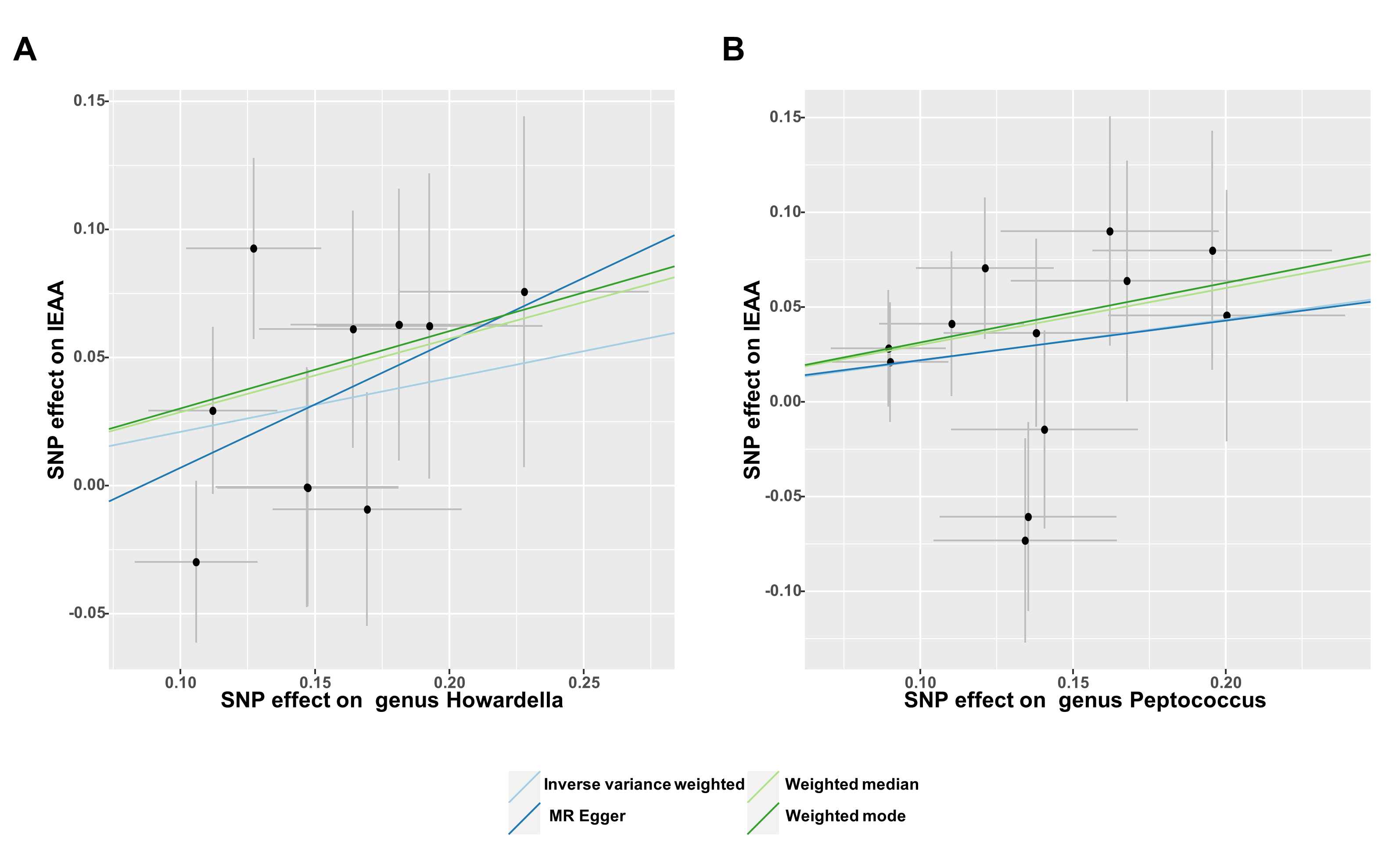


**Supplemental figure S2** Scatter plot for gut microbiota affecting HannumAA risk


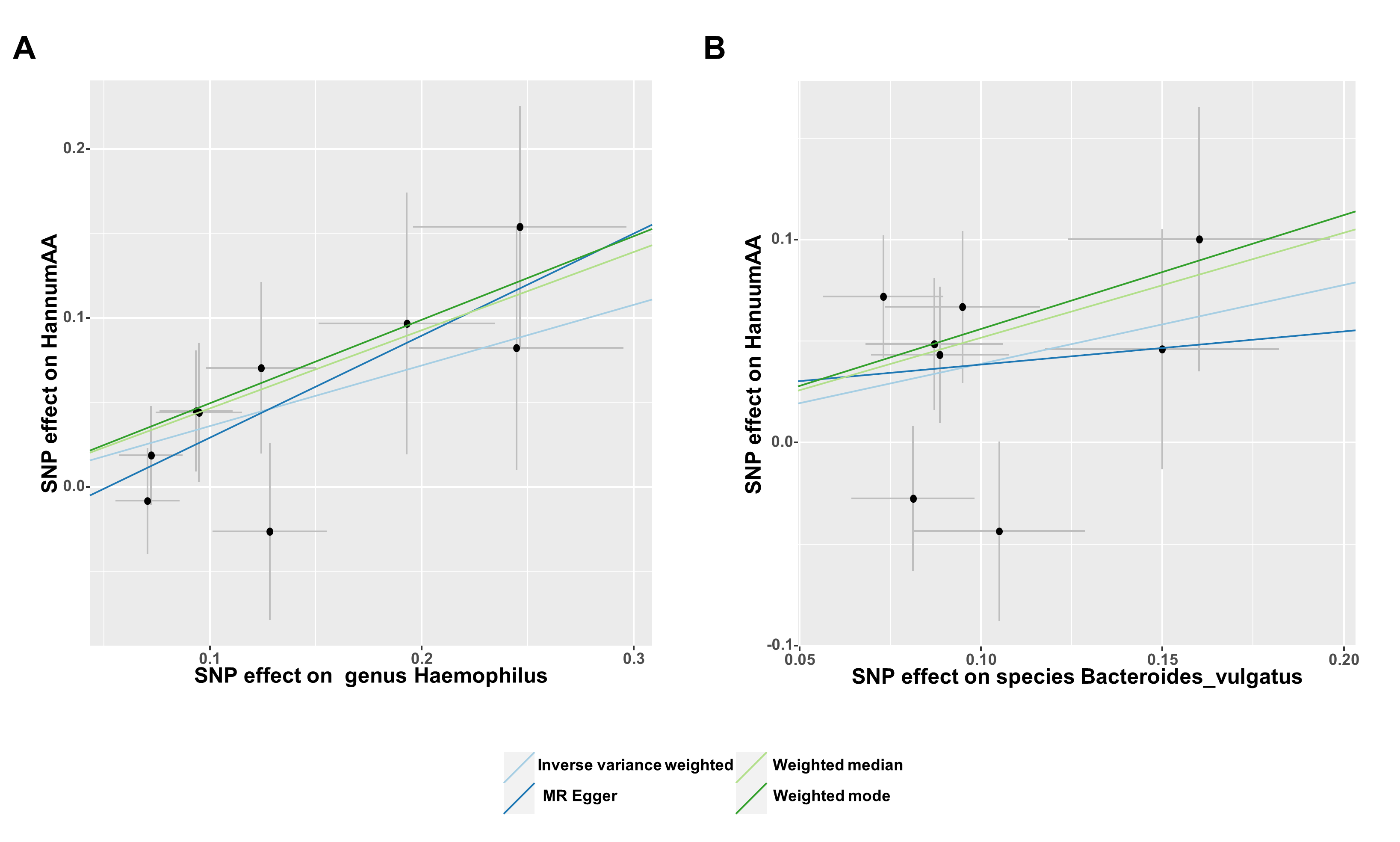


**Supplemental figure S3** Scatter plot for gut microbiota affecting GrimAA risk


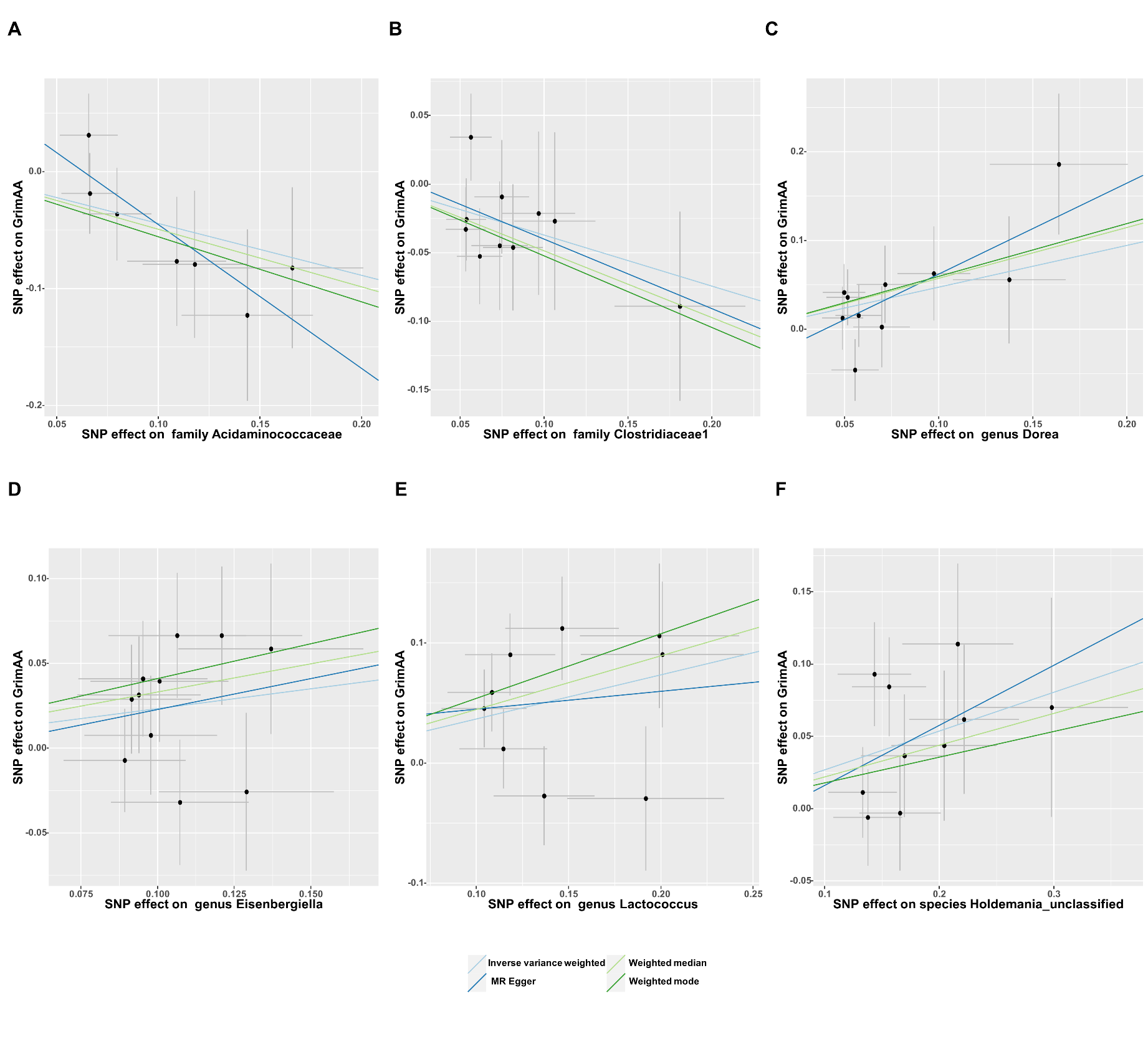


**Supplemental figure S4** Scatter plot for gut microbiota affecting PhenoAA risk


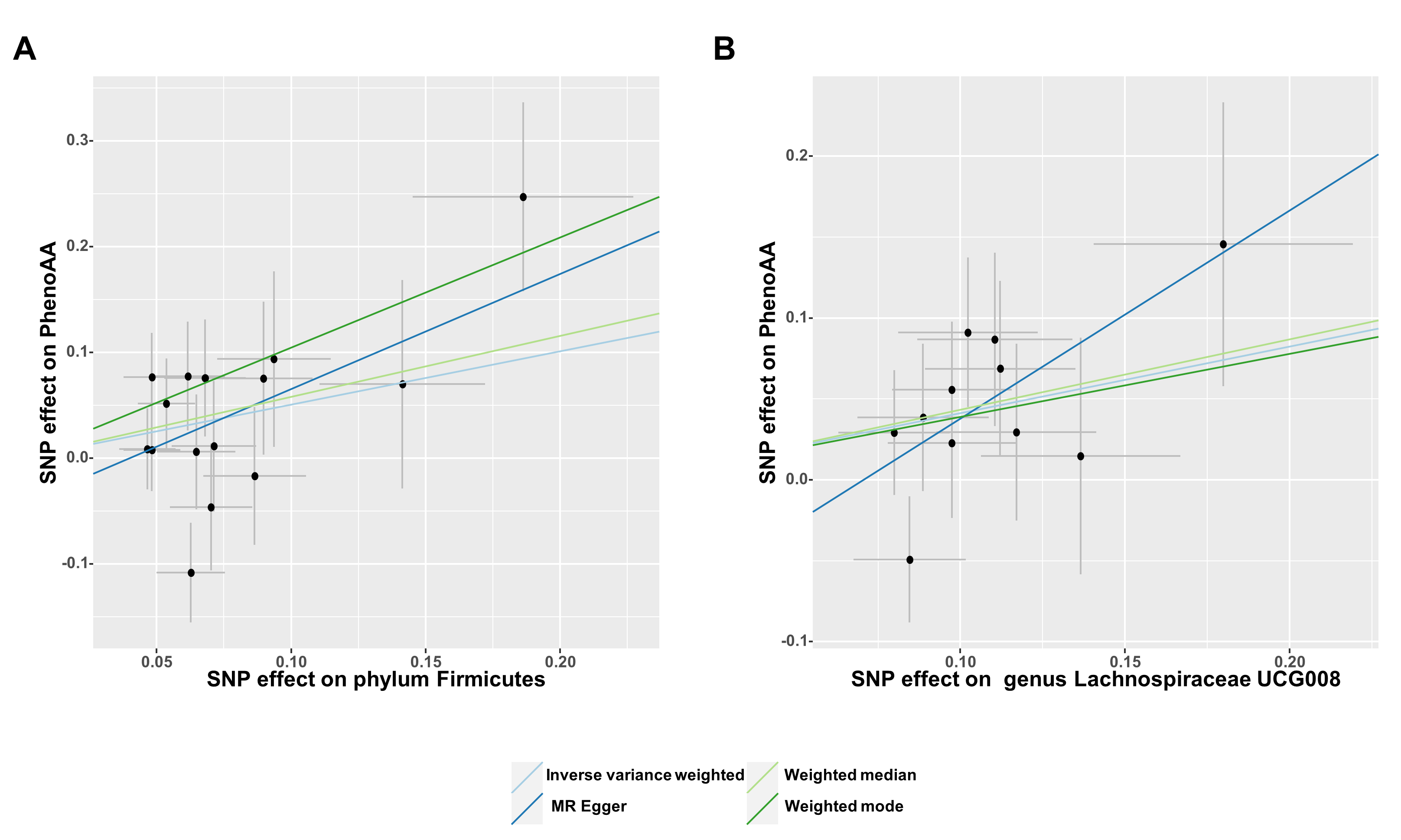


**Supplemental figure S5** Leave-one-out test for gut microbiota affecting IEAA


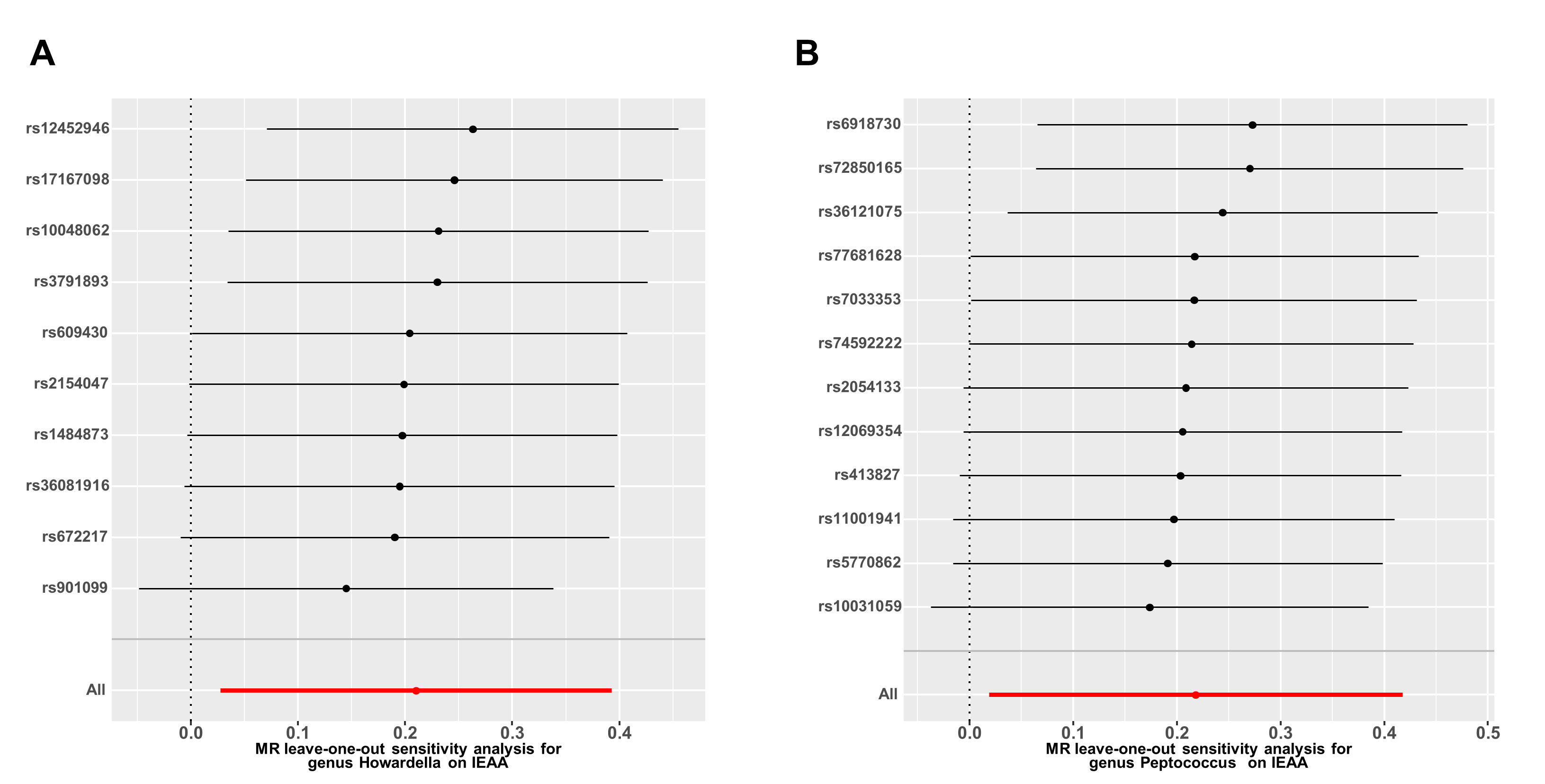


**Supplemental figure S6** Leave-one-out test for gut microbiota affecting HannumAA


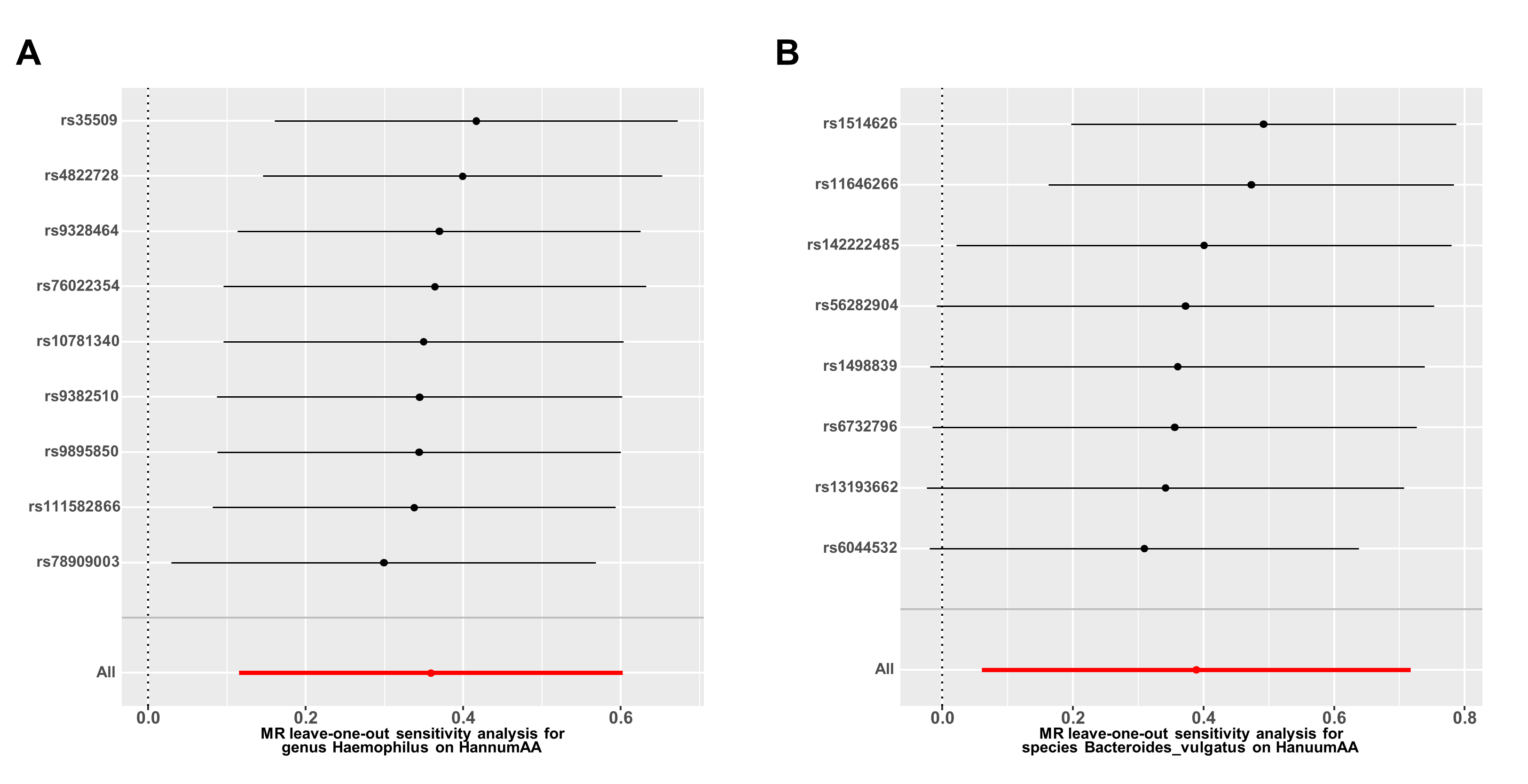


**Supplemental figure S7** Leave-one-out test for gut microbiota affecting GrimAA


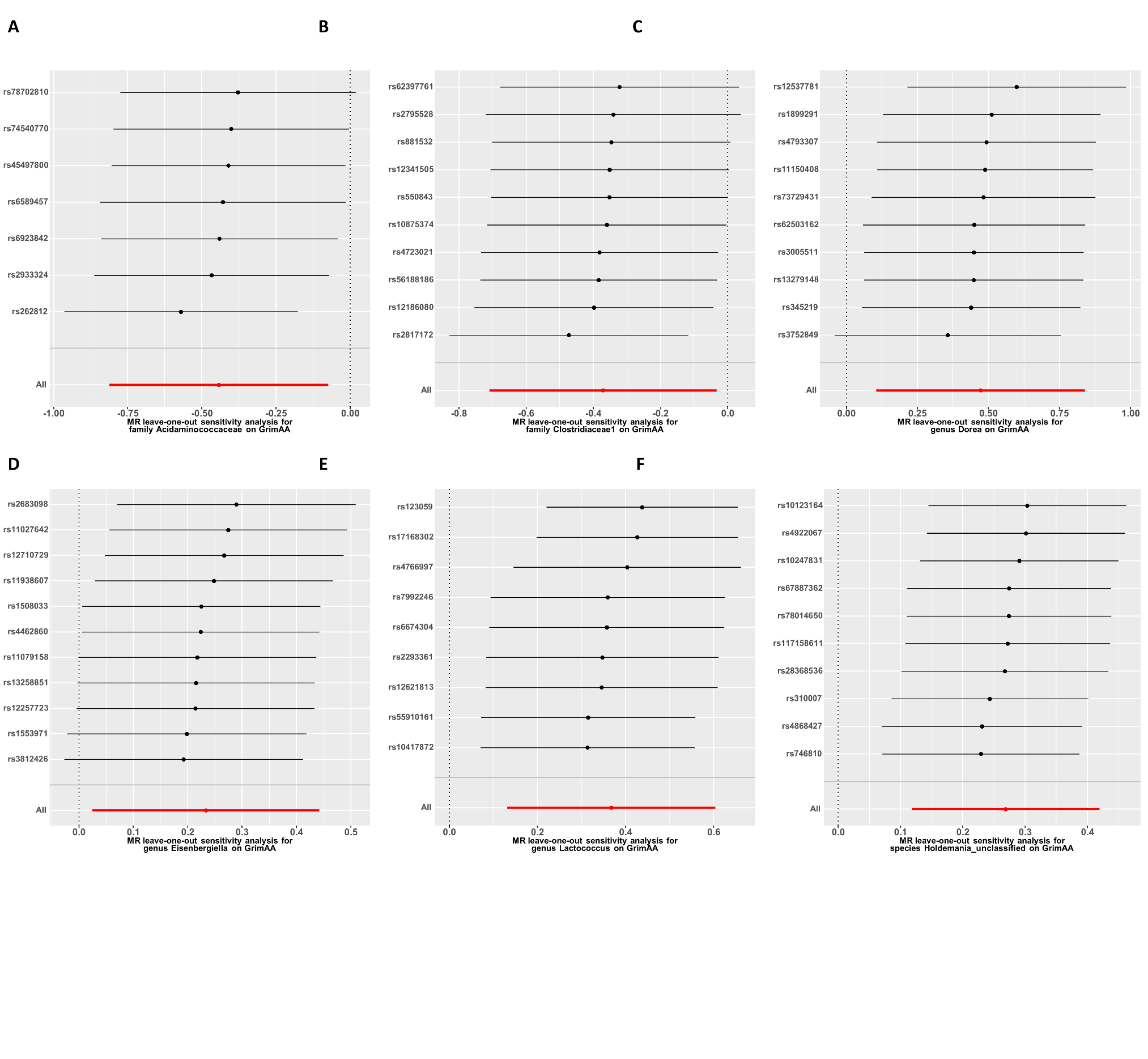


**Supplemental figure S8** Leave-one-out test for gut microbiota affecting PhenoAA


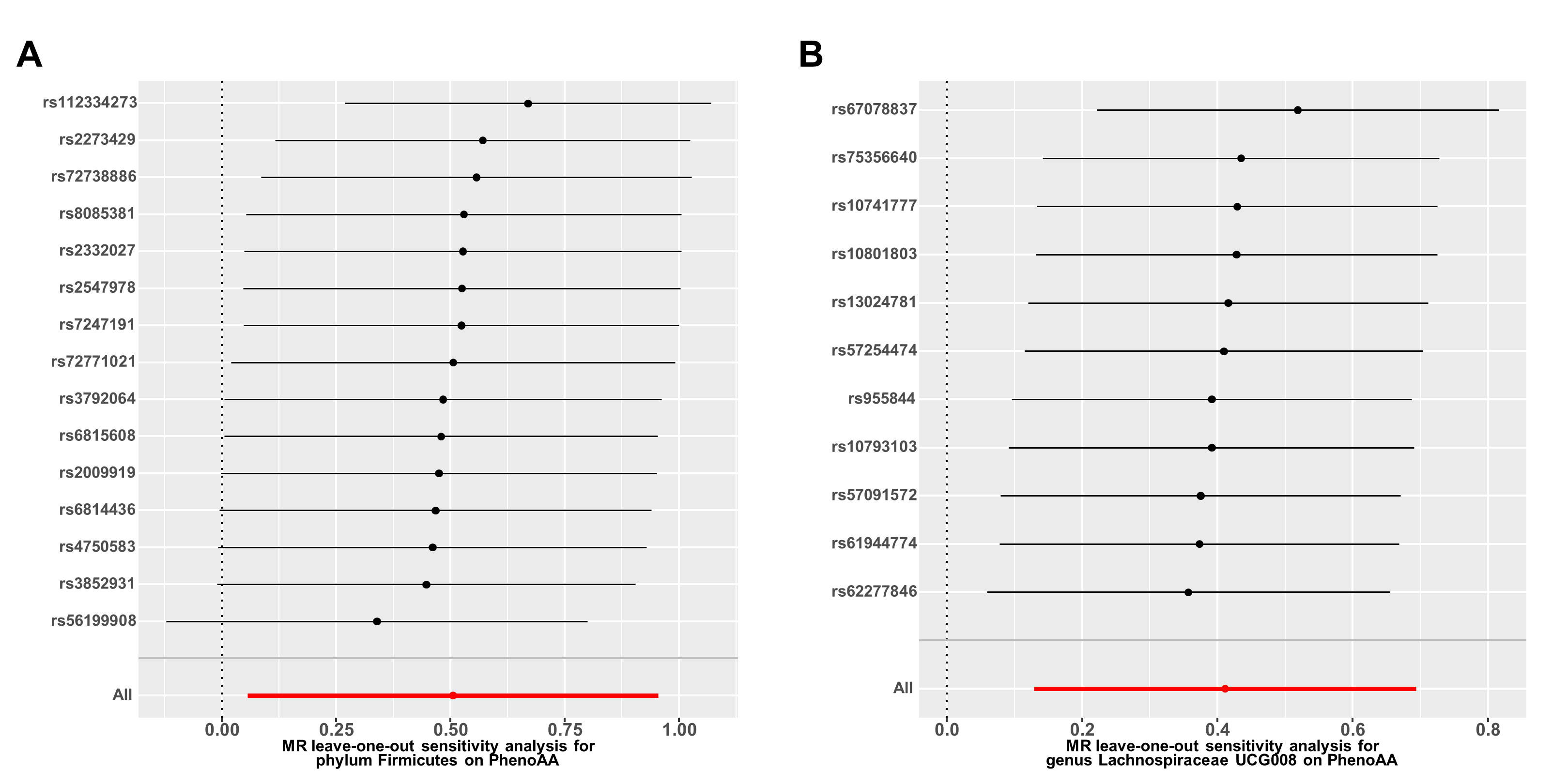

Supplement: Supplementary file 1 — Supplementary Material 1 [file 40520_2024_2877_MOESM1_ESM.docx]
